# High Throughput mRNA Sequencing Reveals Potential Therapeutic Targets of Tao-Hong-Si-Wu Decoction in Experimental Middle Cerebral Artery Occlusion

**DOI:** 10.3389/fphar.2018.01570

**Published:** 2019-01-14

**Authors:** Xianchun Duan, Lan Han, Daiyin Peng, Weidong Chen, Can Peng, Ling Xiao, Qiuyu Bao

**Affiliations:** ^1^The First Affiliated Hospital of Anhui University of Chinese Medicine, Hefei, China; ^2^School of Pharmacy, Anhui University of Chinese Medicine, Hefei, China; ^3^Key Laboratory of Chinese Medicinal Formula Research, Anhui University of Chinese Medicine, Hefei, China; ^4^School of Pharmacy, China Pharmaceutical University, Nanjing, China

**Keywords:** middle cerebral artery occlusion (MCAO), messenger RNA (mRNA), expression profiler, Tao-Hong-Si-Wu decoction, RNA-sequencing, traditional Chinese medicine

## Abstract

**Background:** Experimental and clinical studies have shown that Tao-Hong-Si-Wu decoction (THSWD) improved neurological deficits resulting from Middle Cerebral Artery Occlusion (MCAO). However, the mechanisms of action of THSWD in MCAO have not been characterized. In this study, the mRNA transcriptome was used to study various therapeutic targets of THSWD.

**Methods:** RNA-seq was used to identify differentially expressed genes (DEGs). MCAO-induced up-regulated genes (MCAO vs. control) and THSWD-induced down-regulated genes (compared to MCAO) were identified. Intersection genes were defined as up-regulated differentially expression genes (up-DEGs) identified as MCAO-induced gene expression that were reversed by THSWD. Genes down-regulated by MCAO and up-regulated by THSWD were grouped as another series of intersections. Biological functions and signaling pathways were determined by gene ontology (GO) and Kyoto encyclopedia of genes and genomes (KEGG) pathway analyses. In addition, several identified genes were validated by RT-qPCR.

**Results:** A total of 339 DEGs were filtered based on the 2 series (MCAO vs. control and MCAO vs. THSWD), and were represented by genes involved in cell cycle (rno04110), ECM–receptor interaction (rno04512), complement and coagulation cascades (rno04610), focal adhesion (rno04510), hematopoietic cell lineage (rno04640), neuroactive ligand–receptor interaction (rno04080), cocaine addiction (rno05030), amphetamine addiction (rno05031), nicotine addiction (rno05033), fat digestion and absorption (rno04975), glycerophospholipid metabolism (rno00564), and others. The protein–protein interaction (PPI) network consisted of 202 nodes and 1,700 connections, and identified two main modules by MOCDE.

**Conclusion:** Cell cycle (rno04110), ECM–receptor interaction (rno04512), complement and coagulation cascades (rno04610), focal adhesion (rno04510), hematopoietic cell lineage (rno04640), and neuroactive ligand–receptor interactions (rno04080) are potential therapeutic targets of THSWD in MCAO. This study provided a theoretical basis for THSWD prevention of neurological deficits resulting from intracerebral hemorrhage.

## Introduction

Traditional Chinese medicinal (TCM) herbs have been widely used in China and other Asian countries to combat various diseases for thousands of years. It is extremely important to elucidate the molecular mechanisms of TCM using modern advanced technologies (Efferth et al., 2007; Terrano et al., 2010; Youns et al., 2010). Fortunately, high throughput sequencing-based transcriptome analysis provides an approach for rapidly distinguishing altered mRNAs following drug treatment and thus contributes to understanding and interpretation of mechanisms of action of TCM (Liu and Guo, 2011; Suo et al., 2016). Combination therapy with individual herbs to form specific formulas (Babhulkar, 2009) has been used in TCM for approximately 2,500 years with the goal of increasing therapeutic efficacy and reducing adverse effects (Wang et al., 2008). Wang et al. (2017) used RNA-SEQ to study the pharmacological mechanism of Orexin-A in the treatment of stroke. RNA-seq technique was used to study the expression of transcriptome in rat stroke model treated with Mongolian medicine Eerdun Wurile by Gaowa et al. (2018). Tao-Hong-Si-Wu decoction (THSWD), a formula of TCM herbs, has been used to prevent and treat cerebrovascular diseases in clinical practice in China. This formula contains six commonly used herbs, including Semen prunus (Taoren in Chinese, TR), Flos carthami (Honghua in Chinese, HH), Rehmanniae Radix Praeparata (Shudi in Chinese, SD), Radix *Angelicae sinensis* (Danggui in Chinese, DG), *Paeonia lactiflora* (Baishao in Chinese, BS), and Rhizoma ligustici Chuanxiong (Chuanxiong in Chinese, CX) (Yeh et al., 2007). In previous studies, THSWD protected against cerebral ischemia through PI3K/AKT and Nrf2 signaling pathways (Han et al., 2015). Another study showed that THSWD could regulate angiogenesis and exert neuroprotective activity against cerebral ischemia-reperfusion injury.

In recent decades, the expression of mRNA performed by microarray or high-throughput RNA-seq has been used to reveal molecular mechanisms and explore biomarkers for diagnosis and prediction, especially in complex diseases such as cancer, diabetes and stroke [10–12].

To comprehensively elucidate potential molecular mechanisms, our present study applied an RNA-seq strategy to characterize the key molecular mechanisms of THSWD. At first, we performed RNA-seq in rat brain hemispheres of control, Middle Cerebral Artery Occlusion (MCAO), and THSWD groups. EdgeR and venny were applied to identify up-DEGs and down-DEGs. And then, clusterProfiler, GO, and pathway annotation of up-DEGs and down-DEGs was performed. PPI networks were structured based on STRING database, visualization was carried out using Cytoscape, and modules were identified by MCODE. Finally, key DEGs were verified by qPCR.

## Materials and Methods

### Sample Preparation for UPLC-TOF-MS

The proportion of THSWD compound was TR: HH: SD: DG: BS: CX = 3: 2: 4: 3: 3: 2 (Yeh et al., 2007). Each component was purchased from Anqing Huashi Chinese Herbal Medicine Beverage Co., Ltd. (Anqing City, Anhui Province, China). First, the decoction was extracted with 10× water (10 times the sum of the weight of six components) for 2 h. After filtration, slag was extracted with 8× water (the amount of water added to the total weight of the six components) for 1.5 h. The extract was filtered twice and combined with ethanol to sink conditions. Ethanol containing THSWD at sink conditions was diluted with ethanol, and the solution was filtered using 0.22 μm membranes. 2 μL of filtrate was used for further analysis.

Eleven control compounds were obtained, including gallic acid, rehmannioside D, protocatechuic acid, P-hydroxybenzoic acid, hydroxysafflor yellow A, caffeic acid, amygdalin, chlorogenic acid, paeoniflorin, ferulaic acid, and benzoic acid (Table 1). The 11 control compounds were all provided by the National Institutes for Food. The purity of the products was greater than or equal to 98%.

**Table 1:** Analysis of THSWD by UPLC-TOF-MS.

No.	RT1 (min)	RT2 (min)	Name	Molecular weight	Molecular formula	Source
1	1.72	1.62	Gallic acid	170.12	C_7_H_6_O_5_	HH,BS
2	2.06		Rehmannioside D	686.61	C_27_H_42_O_20_	SD
3	2.69	2.63	Protocatechuic acid	154.12	C_6_H_3_COOH	HH,CX
4	3.80		P-Hydroxybenzoic acid	138.13	C_7_H_6_O_3_	HH
5	4.05	4.06	Hydroxysafflor yellow A	612.53	C_27_H_32_O_16_	HH
6	5.08		Caffeic acid	180.16	C_9_H_8_O_4_	DG,CX
7	5.67	5.66	Amygdalin	457.43	C_20_H_27_NO_11_	TR
8	6.42		Chlorogenic acid	354.31	C_16_H_18_O_9_	DG,HH
9	10.26	10.29	Paeoniflorin	480.45	C_23_H_28_O_11_	BS
10	10.66		Ferulaic acid	194.19	C_10_H_10_O_4_	DG,CX
11	11.41		Benzoic acid	122.12	C_6_H_5_COOH	BS


*RT1 is retention time of mixed reference standard compounds and RT2 is retention time of THSWD*.

### UPLC-QTOF/MS^E^ Analysis

The aqueous extract of THSWD was separated using a Waters Acquity UPLC BEH C18 column (2.1 mm × 100 mm, 1.7 μm, Waters Corporation, Milford, MA, United States). Mobile phases consisted of 0.1% aqueous formic acid (A) and acetonitrile (B). Chromatographic separation was performed at 35°C. Gradient elution with the flow rate of 0.3 mL/min was performed as follows: 3% B at 0–2 min, 3–8% B from 2 to 8 min, 8 to 25% B from 8 to 12 min, 25% B from 12 to 15 min, 25 to 45% B from 15 to 16 min, 45 to 90% B from 16 to 22 min, 90 to 100% B from 22 to 26 min, and 100% B from 26 to 28 min. A Waters SYNAPTG2-Si MS (Waters Corporation, Manchester, United Kingdom) with an ESI source in negative ion mode was used for MS analysis, and leucine enkephalin was used as the accurate mass calibration solution. The desolvation gas temperature was 350°C. The flow rates of the cone and desolvation gases were set at 50 and 600 L/h, respectively. The capillary, cone, and extraction cone voltages were set to –2.5 kV. MS^E^ was used for MS/MS analysis with a low collision energy of 6 V and a high collision energy of 20–80 V. The scan range was *m*/*z* 50–1200. MassLynx^TM^ v4.1 (Waters Co.) and UNIFI^®^Scientific Information System v1.7 (Waters Co.) were used for data acquisition and analysis.

### Animal Experiments and Samples Collection

Male Sprague-Dawley (SD) rats (verified SPF, 180–230 g) were obtained from the Experimental Animal Center of Anhui Medical University. Rats were randomly divided into normal group, MCAO group and THSWD group with 10 rats in each group. All rat anesthetized using sodium pentobarbital during all surgeries to minimize suffering, and preparation of the MCAO model was performed following our previous study (Han et al., 2015), which also conformed to the NIH Guide for animal care and welfare (NIH Publications No. 80-23). The procedures of THSWD extraction and medicine administration were described in the material and methods section “Sample Preparation for UPLC-TOF-MS” and our previous research (Han et al., 2015). Four days after MCAO, the left hemispheres were collected and immediately frozen in liquid nitrogen. The study protocol was approved by the Committee on the Ethics of Animal Experiments of Anhui University of Chinese Medicine (2012AH-036-03).

### RNA Extraction and RNA-Sequencing

Total RNA was extracted using mirVana^TM^ miRNA Isolation kit (Cat# AM1561, Ambion) following the manufacturer’s instructions. Quantity and quality of RNA samples were determined using a NanoDrop 2000 (Thermo Fisher Scientific, Wilmington, DE, United States) and Agilent 2100 Bioanalyzer (Agilent Technologies, United States). TruSeq^®^Stranded Total RNA Sample Preparation kit was used to prepare libraries following the manufacturer’s instructions, and purified libraries were quantified using a Qubit 2.0^®^Fluorometer (Life Technologies, United States) and Agilent 2100 Bioanalyzer (Agilent Technologies, United States). Clusters were generated by cBot library and sequenced using an Illumina HiSeq 2500 (Illumina, United States). Sequencing was performed at Origin-Biotech Inc. (Ao-Ji Biotech, Shanghai, China).

### Differential Expression Genes Analysis

FastQC was conducted for Quality control (QC) of RNA-Seq reads (version. 0.11.3)^1^. Trimming was performed by seqtk for known Illumina TruSeq adapter sequences, poor reads, and ribosomal RNA reads^2^. The trimmed reads were then mapped to the *Rattus norvegicus* reference genome (rno6) using Hisat2 (version: 2.0.4) (Kim et al., 2015; Pertea et al., 2016). Stringtie (version: 1.3.0) was performed for each gene count from trimmed reads (Pertea et al., 2015, 2016). Gene counts were normalized by TMM (Robinson and Oshlack, 2010), and FPKM was performed using Perl script (Mortazavi et al., 2008). EdgeR was used to determine differential gene expression (Robinson et al., 2010), with significance determined by a *p*-value < 0.05 and absolute value of a log 2-fold change > 1 (Benjamini et al., 2001). Venny was applied to screen up-DEGs (MCAO vs. Control and THSWD vs. MCAO) and down-DEGs (MCAO vs. Control and THSWD vs. MCAO).

### Functional Enrichment Analysis

Gene Ontology (GO) and Kyoto Encyclopedia of Genes and Genomes (KEGG) pathways were enriched using R package to better understand the functions of DEGs (Altermann and Klaenhammer, 2005). In our study, clusterProfiler was applied to analysis of GO terms and KEGG pathways, and top30 GOs and pathway were presented (Ashburner et al., 2000).

### PPI Network Construction and Module Analysis

STRING is a database that provides comprehensive information about interactions between proteins, including prediction and experimental interaction data (Szklarczyk et al., 2011). In our study, the STRING tool was used to generate PPIs among the DEGs, based on interactions with combined scores ≥ 0.4. Then, Cytoscape was used to visualize the network (Shannon et al., 2003). The PPI network was used to filter modules based on the Molecular Complex Detection plug-in (MCOD) in Cytoscape (Bader and Hogue, 2003) with the following conditions: degree cut-off = 2, k-core = 2, node score cut-off = 0.2, and max depth = 100. MCODE score ≥ 4 and node ≥ 10 were considered for functional enrichment analysis of the modules. Moreover, GO and KEGG enrichment for DEGs in the four modules was performed using clusterProfiler.

### Validation of Differentially Expressed mRNAs From the Sequencing Profile by qRT-PCR

Real-time PCR was performed to verify the RNA-seq results, and GAPDH was used as the internal control. Relative expression of mRNAs was determined using the 2^-ΔΔCT^ method. The following six genes were analyzed: Cdk1, Ccna2, Cdc20, Ndc80, Th, and Calb2. Brain tissues from control, MCAO, and THSWD groups were analyzed (15 samples).

### Statistical Analysis

Raw data are shown as mean ± standard deviation (SD). Statistical calculations were carried out using GraphPad Prism 5 (GraphPad Software, La Jolla, CA, United States). A *p*-value less than 0.05 was considered to be statistically significant.

## Results

### Identification of Chemical Components of THSWD

**FIGURE 1:**
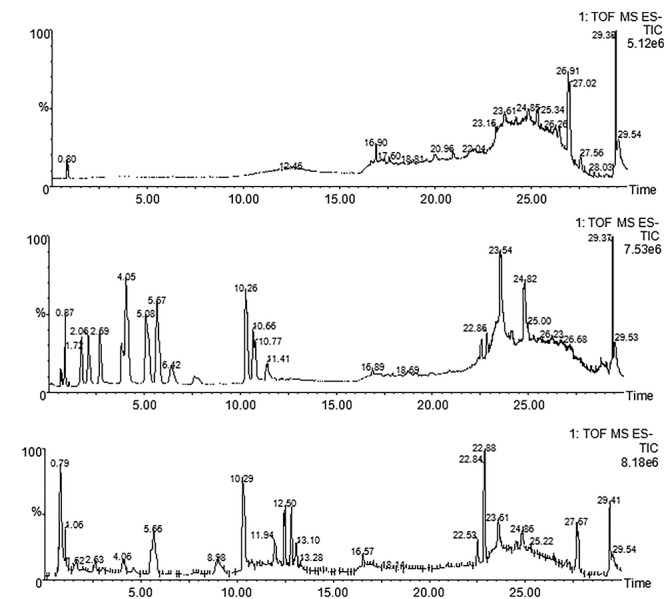
UPLC-TOF-MS analysis of THSWD. Chromatographic fingerprints of control **(A)**, standard components **(B)** and THSWD **(C)** by UPLC-TOF-MS in negative ion mode.

Using UPLC-TOF-MS, we have tentatively identified five components: gallic acid (**t_R_**≈1.72 min, MW = 170.12), protocatechuic acid (**t_R_**≈2.69 min, MW = 154.12), hydroxysafflor yellow A (**t_R_**≈4.05 min, MW = 612.53), amygdalin (**t_R_**≈5.67, MW = 457.43), and paeoniflorin (**t_R_**≈10.26 min, MW = 480.45) by comparing their fingerprints with standards (Figure 1 and Table 1). These components were sourced from HH, BS, CX, and TR (Table 1).

### Identification of DEGs

**FIGURE 2:**
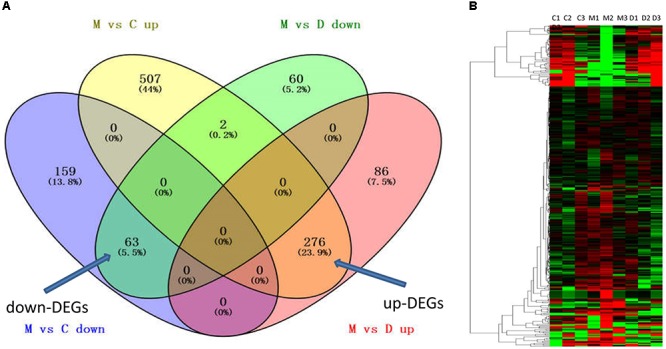
THSWD reversed DEGs with MCAO. **(A)** The intersection gene of down-DEGs (MCAO vs. Control and THSWD vs. MCAO, down- and up-regulated, respectively) and up-DEGs (MCAO vs. Control and THSWD vs. MCAO, up- and down-regulated, respectively). **(B)** Heatmap of down-DEGs and up-DEGS (339 genes total). C (or N) refers to the control group, M refers to the MCAO group, D refers to the THSWD treatment group.

To further understand the multifaceted mechanisms of THSWD in MCAO, we performed RNA-seq to obtain the transcriptomes of control, MCAO, and MCAO+THSWD samples. Using bioinformatics analysis, 339 DEGs were filtered (MCAO vs. control and MCAO vs. THSWD, |log2(fold-change)| > 1 and *p* < 0.05). Figure 2 and Supplementary Table S1 show that 63 down-DEGs were screened from the intersection of 222 down-regulated mRNAs (MCAO vs. Control) and 125 down-regulated mRNAs (MCAO vs. THSWD) (Figure 1 and Supplementary Table S1). In addition, 276 up-DEGs were identified from the intersection of 362 up-regulated mRNAs (MCAO vs. Control) and 785 up-regulated mRNAs (MCAO vs. THSWD) (Figure 2 and Supplementary Table S1).

### GO Analysis of DEGs Altered by THSWD

To further understand the DEGs associated with THSWD treatment of MCAO, GO enrichment analysis was performed with 63 down-DEGs and 276 up-DEGS by clusterProfiler.

**FIGURE 3:**
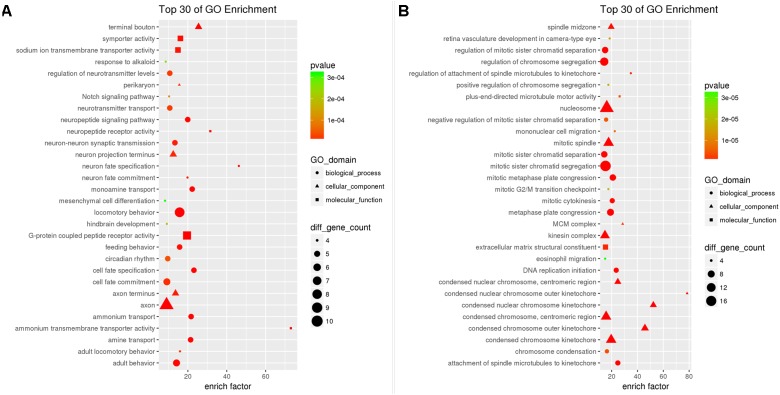
Results of Gene Ontology enrichment analysis for down-DEGs reversed by THSWD **(A)** and down-DEGs **(B)** resulting from MCAO.

Enrichment analysis showed that a total of 196 GO-terms were significantly enriched with 63 down-DEGs, 30 of which were BP, 40 of which were CC, and 60 of which were MF with *p* < 0.05. The top10 enriched GO biological processes were locomotor behavior (GO:0007626), neuron fate specification (GO:0048665), behavior (GO:0007610), cell fate specification (GO:0001708), monoamine transport (GO:0015844), ammonium transport (GO:0015696), amine transport (GO:0015837), neuropeptide signaling pathway (GO:0007218), adult behavior (GO:0030534), and single-organism behavior (GO:0044708). Neuron projection (GO:0043005), axon (GO:0030424), terminal bouton (GO:0043195), cell projection (GO:0042995), synapse (GO:0045202), neuronal cell body (GO:0043025), axon terminus (GO:0043679), cell body (GO:0044297), neuron projection terminus (GO:0044306), and perikaryon (GO:0043204) were the top10 enriched cellular components. The enriched GO molecular functions were G-protein coupled peptide receptor activity (GO:0008528), ammonium transmembrane transporter activity (GO:0008519), neuropeptide receptor activity (GO:0008188), symporter activity (GO:0015293), sodium ion transmembrane transporter activity (GO:0015081), secondary active transmembrane transporter activity (GO:0015291), ion transmembrane transporter activity (GO:0015075), substrate-specific transmembrane transporter activity (GO:0022891), cation transmembrane transporter activity (GO:0008324), and transmembrane transporter activity (GO:0022857). The top30 GO-terms with highest enrichment factor are shown in Figure 3A.

The 276 up-DEGs were significantly enriched into 619 GO-terms, 482 of which were BP, 61 of which were CC, and 54 of MF with *p* < 0.05. The top10 enriched GO biological processes were mitotic cell cycle process (GO:1903047), mitotic cell cycle (GO:0000278), mitosis (GO:0007067), cell division (GO:0051301), chromosome segregation (GO:0007059), cell cycle (GO:0007049), mitotic sister chromatid segregation (GO:0000070), sister chromatid segregation (GO:0000819), chromosome organization (GO:0051276), and regulation of cell cycle process (GO:0010564). Meanwhile, we found that many neuron death related GO-terms were significantly enriched such as negative regulation of neuron apoptotic process (GO: 0043524, *p* = 5.120e-03), negative regulation of neuron death (GO:1901215, *p* = 1.598e-02), and regulation of neuron apoptotic process (GO:0043523, *p* = 3.748e-02). Chromosome (GO:0005694), nucleosome (GO:0000786), spindle (GO:0005819), protein-DNA complex (GO:0032993), chromosome, centromeric region (GO:0000775), nuclear chromosome (GO:0000228), kinetochore (GO:0000776), condensed chromosome kinetochore (GO:0000777), chromatin (GO:0000785), and microtubule cytoskeleton (GO:0015630) were the top10 enriched cellular components. The enriched GO molecular functions were protein binding (GO:0005515), protein heterodimerization activity (GO:0046982), microtubule motor activity (GO:0003777), microtubule binding (GO:0008017), protein dimerization activity (GO:0046983), motor activity (GO:0003774), tubulin binding (GO:0015631), extracellular matrix structural constituent (GO:0005201), cytoskeletal protein binding (GO:0008092), and protein complex binding (GO:0032403). The top30 GO-terms with highest enrichment factors are shown in Figure 3B.

### KEGG Analysis of DEGS Altered by THSWD

Pathway enrichment analysis could provide further information regarding functions of genes and their interactions. ClusterProfiler was applied to GO enrichment analysis with 63 down-DEGs and 276 up-DEGs, which were associated with THSWD treatment of MCAO.

**FIGURE 4:**
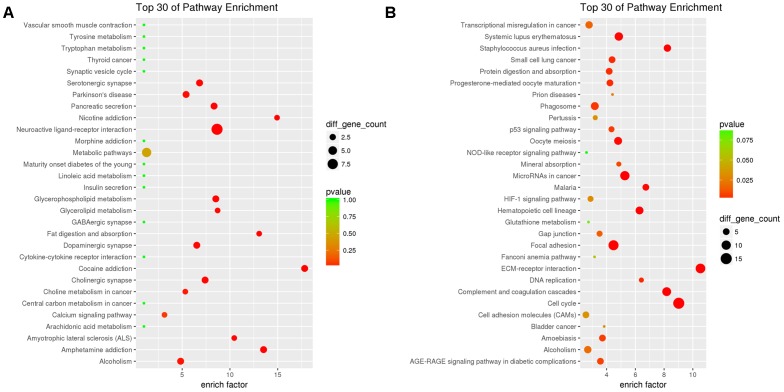
Results of KEGG enrichment analysis for down-DEGs reversed by THSWD **(A)** and down-DEGs **(B)** following MCAO.

KEGG enrichment analysis showed that a total of 45 pathway terms were enriched with 63 down-DEGs, 15 of which were significant with *p* < 0.05. The top10 enriched pathways were neuroactive ligand–receptor interaction (rno04080), cocaine addiction (rno05030), amphetamine addiction (rno05031), nicotine addiction (rno05033), fat digestion and absorption (rno04975), glycerophospholipid metabolism (rno00564), pancreatic secretion (rno04972), cholinergic synapse (rno04725), and amyotrophic lateral sclerosis (ALS) (rno05014). The top30 pathway-terms with highest enrichment factors are shown in Figure 4A.

We performed KEGG pathway enrichment analysis for up-DEGs, and found 136 pathway terms including 29 pathway terms with *p*-value < 0.05. Top10 pathways with the greatest enrichment were cell cycle (rno04110), ECM–receptor interaction (rno04512), complement and coagulation cascades (rno04610), microRNAs in cancer (rno05206), focal adhesion (rno04510), *Staphylococcus aureus* infection (rno05150), hematopoietic cell lineage (rno04640), systemic lupus erythematosus (rno05322), malaria (rno05144), and oocyte meiosis (rno04114). The top30 enriched pathways are presented in Figure 4B.

### PPI Network

**FIGURE 5:**
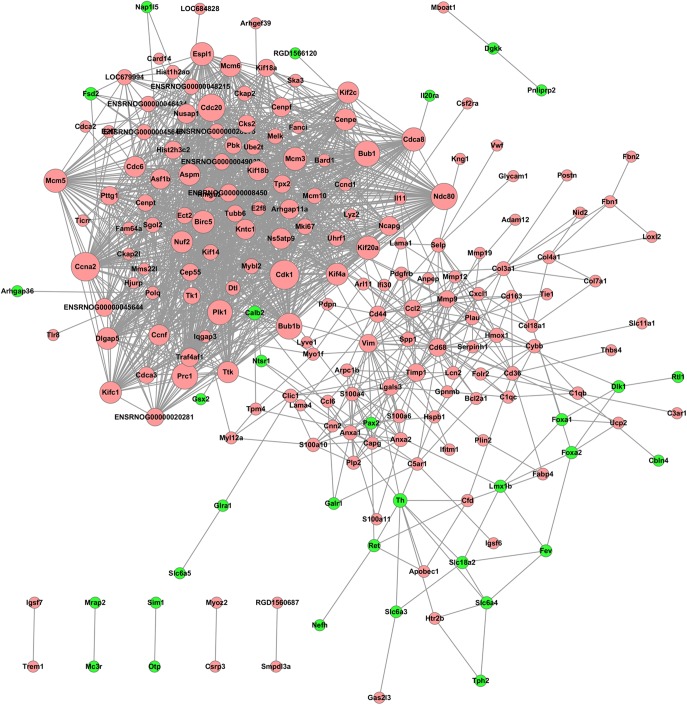
PPI network of the DEGs resulting from THSWD treatment of MCAO. Node sizes correlate with node importance; Pink nodes denote up-regulated genes, while green nodes denote down-regulated genes; PPI, protein–protein interaction; DEGs, differentially expressed genes.

Significant DEGs were used to construct a PPI network based on the STRING database. The PPI network consisted of 202 nodes and 1,700 interactions, as shown in Figure 5. Dozens of gene nodes were high in connectivity degrees, such as Cdk1 (cyclin-dependent kinase 1, degree = 66), Ccna2 (cyclin A2, degree = 64), Cdc20 (cell division cycle 20, degree = 59), Ndc80 (NDC80 kinetochore complex component, degree = 56), Bub1 (BUB1 mitotic checkpoint serine/threonine kinase, degree = 54), Bub1b (BUB1 mitotic checkpoint serine/threonine kinase B, degree = 54), Prc1 (protein regulator of cytokinesis 1, degree = 54), Plk1 (polo-like kinase 1, degree = 50), Th (tyrosine hydroxylase, degree = 10), and Calb2 (calbindin 2, degree = 10). Top10 down-DEGs and up-DEGs with degree ≥ 5 are listed in Table 2.

**Table 2:** Top10 degrees of up- and down-regulated genes (node degree ≥ 5).

		MCAO vs. Control		MCAO vs. THSWD			
gene_id	gene_ name	log2FC *p*-value		log2FC *p*-value		Up/down	Degree
ENSRNOG00000013133	Foxa2	-∞	0.018601	-∞	0.000756	DOWN	5
ENSRNOG00000017019	Lmx1b	-∞	0.003147	-∞	0.002052	DOWN	5
ENSRNOG00000014751	Ret	-1.7584	0.004092	-1.49327	0.019153	DOWN	5
ENSRNOG00000008890	Slc18a2	-4.44319	0.001064	-3.97981	0.00099	DOWN	5
ENSRNOG00000003476	Slc6a4	-6.84749	0.000207	-5.45886	5.65E-05	DOWN	5
ENSRNOG00000019278	Fsd2	-3.20499	0.004022	-3.09428	0.005197	DOWN	6
ENSRNOG00000016977	Calb2	-2.19721	0.005492	-1.92923	0.022153	DOWN	10
ENSRNOG00000020410	Th	-4.99003	0.000109	-5.12072	2.13E-05	DOWN	10
ENSRNOG00000031431	Cdca8	2.794596	0.005599	1.979186	0.032353	UP	45
ENSRNOG00000024428	Kif20a	5.474395	0.000142	3.496781	0.006566	UP	45
ENSRNOG00000014336	Mcm5	3.454364	0.000825	2.705457	0.008317	UP	45
ENSRNOG00000018815	Plk1	2.267746	0.013009	2.222583	0.015802	UP	50
ENSRNOG00000032778	Bub1	3.512779	0.000765	2.440496	0.01259	UP	54
ENSRNOG00000007906	Bub1b	5.783106	2.13E-05	3.628812	0.001801	UP	54
ENSRNOG00000013057	Prc1	2.479756	0.014561	2.113974	0.016491	UP	54
ENSRNOG00000013727	Ndc80	3.562942	0.005167	2.669483	0.026257	UP	56
ENSRNOG00000028415	Cdc20	2.615553	0.007635	2.791868	0.00509	UP	59
ENSRNOG00000015423	Ccna2	4.844517	4.87E-05	3.348336	0.002291	UP	64
ENSRNOG00000000632	Cdk1	4.649292	0.000415	4.042394	0.001316	UP	66


**FIGURE 6:**
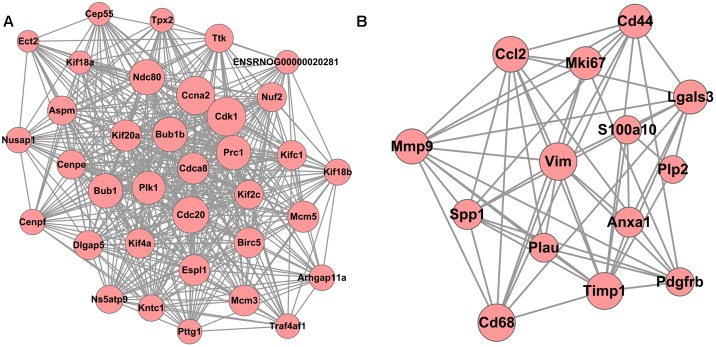
PPI Network of DEGs reversed by THSWD involved in Modules 1 **(A)** and 2 **(B)**. Pink nodes denote up-regulated genes; PPI, protein–protein interaction; DEGs, differentially expressed genes.

A total of 10 modules were obtained using default criteria by MCODE. Modules were listed in descending order by MCODE score. Two modules with MCODE score ≥ 3 and nodes ≥ 10 were named as module 1 and module 2. Two modules were selected for module network visualization (Figure 6). Most DEGs belonging to these modules were up-regulated in the MCAO model group in both modules.

### The Results of qRT-PCR Verification of the DEGs

**FIGURE 7:**
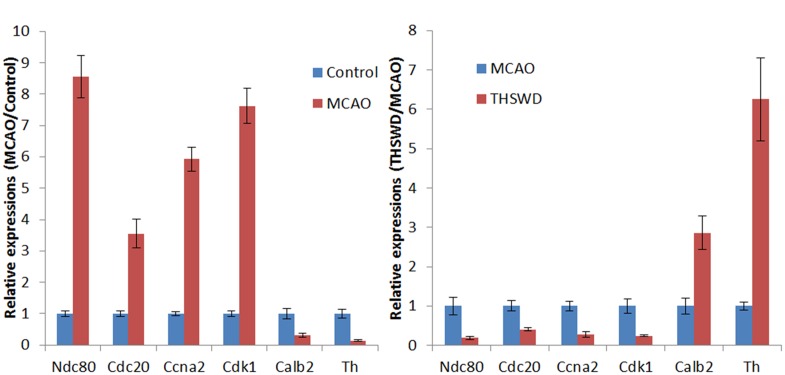
Verification of DEGs by qRT-PCR. Expression of six genes in brain tissues was detected by qRT-PCR, and shown by the expression fold changes. GAPDH was used as the internal control. **(A)** presents the results of MCAO vs control, **(B)** presents the results of THSWD vs MCAO.

As shown in Figure 7, real-time PCR results showed that Cdk1, Ccna2, Cdc20, and Ndc80 were over-expressed in the MCAO group. Meanwhile, Th and Calb2 showed low expression in the MCAO group as determined by real-time PCR and RNA-seq (Figure 7A). THSWD reversed expression of these differentially expressed genes (DEGs) to different degrees in MCAO (Figure 7B). These results verified that the RNA-seq results were consistent with real-time PCR results, and the RNA-seq results were reliable. Primers are listed in Table 3.

**Table 3:** Primer sequences.

Gene	Primers sequences	PCR product length (bp)
GAPDH	F: CCTGGTATGACAACGAATTTG	131
	R: CAGTGAGGGTCTCTCTCTTCC	
Cdc20	F: GCACCAGTAATGCTGAGGTG	128
	R: TCGTGAACCACTTGACAGGA	
Cdk1	F: GTACGGCAATCCGGGAAATC	98
	R: GAGATACAGCCTGGAGTCCT	
Ccna2	F: AGCTCTCTACACAGTCACAGG	106
	R: GGTCTGGTGAAGGTCCATGA	
Ndc80	F: GCAAGAGGGCTGTGAGAATG	140
	R: CAGCAGGTGCTTGTGTTTCT	
Th	F: GTCGGAAGCTGATTGCAGAG	129
	R: TAGCATAGAGGCCCTTCAGC	
Calb2	F: TGACACAGACAGAAGTGGCT	127
	R: CCGTAGTATGGTCTGGGTGT	


## Discussion

We analyzed the protein-coding mRNA expression profile in cerebral hemispheres from experimental and control rats by high throughput RNA-seq. 1,007 DEGs were identified, and GO, KEGG pathway, PPI, and PPI module analyses were performed, providing a better understanding of the pathological mechanisms of MCAO.

### Cell Cycle

**FIGURE 8:**
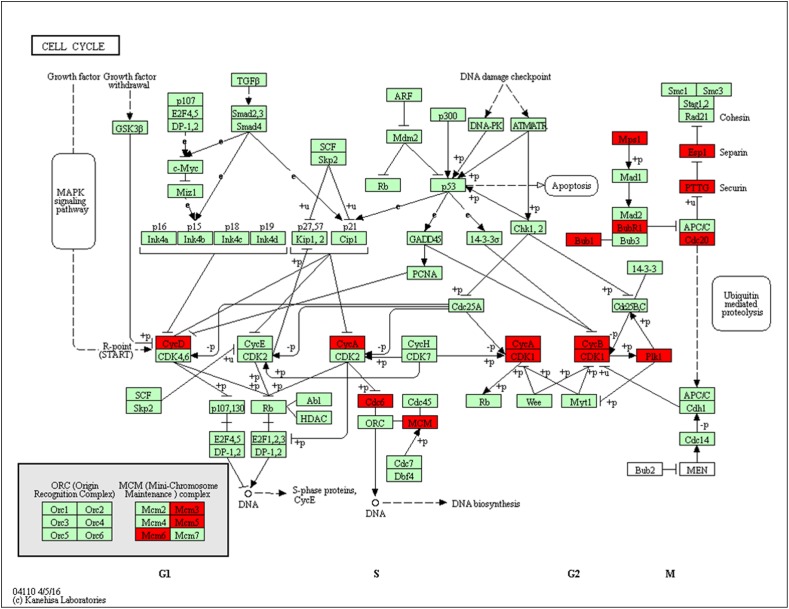
Significantly enriched KEGG pathways in the cell cycle. Up-DEGs are marked in red. The pictures were drawn by KEGG Mapper (www.kegg.jp/kegg/tool/map_pathway2.html).

Using pathway enrichment analysis, 15 DEGs were enriched to cell cycle: Cdk1, Cdc20, Cdc6, Ttk, Ccnb1, Bub1, Mcm6, Pttg1, Bub1b, Mcm3, Espl1, Mcm5, Ccna2, Plk1, and Ccnd1 (Figure 8). Nearly all of these DEGs were up-regulated in MCAO. THSWD reversed MCAO-induced upregulation. Following Cyclin A (Ccna2) or B (Ccnb1) binding and activation of cyclin-dependent kinase 1 (Cdk1), Cdk1 phosphorylates key substrates, resulting in G2-phase arrest, M-phase, and promotion of cytokinesis. Previous studies showed that activation of Cdk1 was involved in neuronal death by phosphorylation of Bad27, a pro-apoptotic protein, or inhibition of phosphorylation of Bcl-xL, Bcl-2, and Mcl-1 which are anti-apoptotic proteins (Harley et al., 2010; Terrano et al., 2010). Moreover, some studies showed similar results in transient ischemia (Wen et al., 2004; Terrano et al., 2010). Besides the Bcl-2 family, Cdk1 also phosphorylates the transcription factor FOXO1, which leads to cell death (Wen et al., 2004). We presumed that during MCAO, Cdk1 induced ischemic neuronal death via the Bcl2 family or FOXO1 promoting signaling pathway, and THSWD protected against MCAO damage by inhibiting these pathways.

### Complement and Coagulation Cascades

**FIGURE 9:**
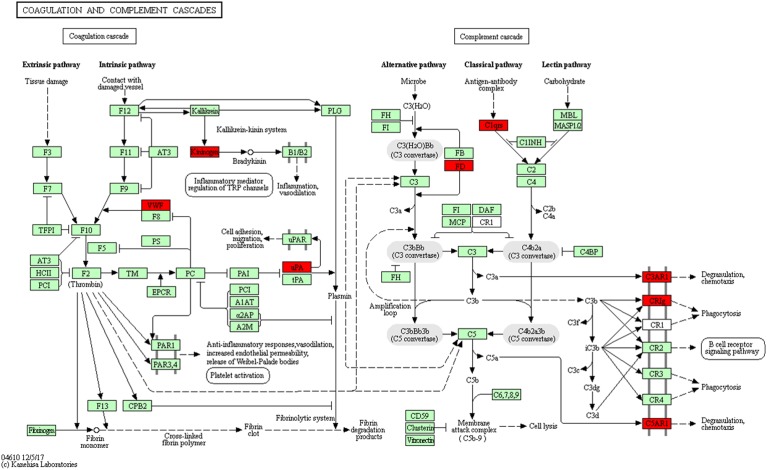
Significantly enriched KEGG pathways in complement and coagulation cascades. The up-DEGs are marked in red. The pictures were drawn by KEGG Mapper (www.kegg.jp/kegg/tool/map_pathway2.html).

Using pathway enrichment analysis, 10 DEGs were enriched into the complement and coagulation cascades, such as C3ar1, Plau, C1qb, C1qc, Vwf, Kng1, Cfd, Vsig4, and C5ar1 (Figure 9). Previous studies reported that complement components C3 and C5 can promote MCAO injury by complement cascade and TLR2/NFκB activation (Conductier et al., 2010; Semple et al., 2010; Xu et al., 2017). Activation of complement and coagulation cascades result in an inflammatory response, which includes degranulation, chemotaxis, and phagocytosis. We found that there were 78 abnormally expression genes in MCAO involved in the inflammatory response. THSWD treatment reversed abnormal expression of 18 of the 78 genes. Our study confirmed these findings, and THSWD inhibited C3ar1, C1qb, C1qc, and C5ar1 expression changes resulting from MCAO. Therefore, we speculated that THSWD can reverse MCAO injury by inhibiting complement and coagulation cascades.

### Neuroactive Ligand–Receptor Interaction

**FIGURE 10:**
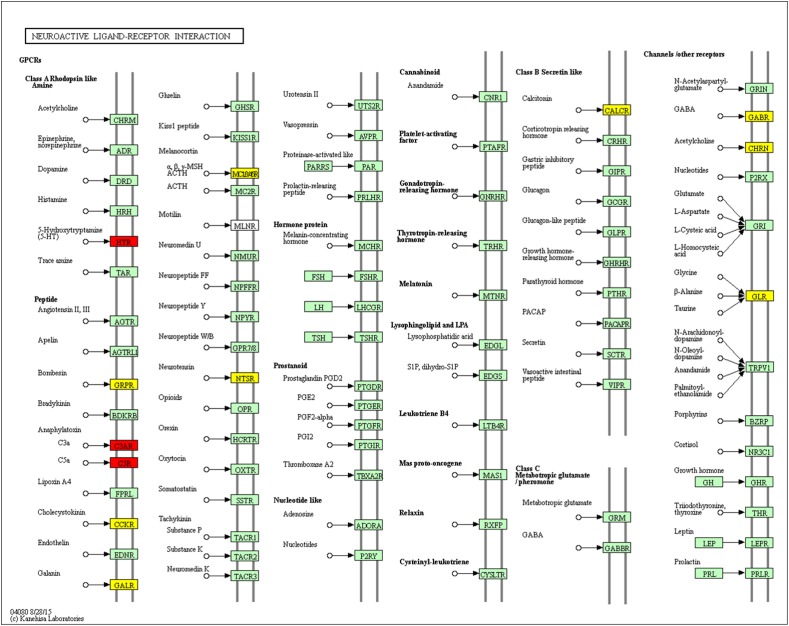
Significantly enriched KEGG pathways in neuroactive ligand–receptor interactions. Up-DEGs are marked in red. Down-DEGs are marked with yellow. The pictures were drawn by KEGG Mapper (www.kegg.jp/kegg/tool/map_pathway2.html).

Using pathway enrichment analysis, we found 12 DEGs involved in neuroactive ligand–receptor interactions: Brs3, Mc3r, Calcr, Chrna6, Glra1, Galr1, Ntsr1, Cckar, Gabre, C3ar1, Htr2b, and C5ar1 (Figure 10). Lan et al. (2014) found that 5-HT and 5-HT1A receptor expression were suppressed in rats with MCAO, and treadmill exercise could blunt this effect. We found that Htr2a and htr2b were up-regulated in the MCAO group, and Htr2b was suppressed by THSWD. We hypothesized that MCAO could induce htr2a and htr2b overexpression, leading to excitability during the early stage of MCAO, and that THSWD could reverse this dysregulation. Previous studies have shown that GABA-A and glycine receptor expression were suppressed in rats with MCAO, and receptor agonists could reverse this effect (Wang et al., 2007; Costa et al., 2016). We found that some subunits of GABA-B, such as Gabrr3, Gabrq, Gabra6, Gabre, and Gabrr1 were dysregulated during MCAO, and Gabre was modulated by THSWD.

## Author Contributions

XD and DP conceived and designed the study. XD and LH performed the UPLC-QTOF/MS^E^ analysis. XD, LX, and QB performed the animal experiments. XD, WC, and CP analyzed the data and pre-viewed the manuscript. All authors read and approved the final manuscript.

## Conflict of Interest Statement

The authors declare that the research was conducted in the absence of any commercial or financial relationships that could be construed as a potential conflict of interest.

## Footnotes

1 http://www.bioinformatics.babraham.ac.uk/projects/fastqc

2 https://github.com/lh3/seqtk

## Supplementary Material

TABLE S1 List of all differentially expressed genes.
